# Prevertebral abscess associated with meningitis: Double cause of neck stiffness

**DOI:** 10.1016/j.idcr.2022.e01549

**Published:** 2022-06-28

**Authors:** Hidenori Higashi, Ken-ichiro Kobayashi, Anna Eto, Tadahiro Goto

**Affiliations:** a Department of Emergency and Critical Care Medicine, Japanese Red Cross Wakayama Medical Center, Wakayama, Japan; b Department of Infectious Diseases, Japanese Red Cross Wakayama Medical Center, Wakayama, Japan; c Department of Otolaryngology, Japanese Red Cross Wakayama Medical Center, Wakayama, Japan; d Department of Clinical Epidemiology and Health Economics, School of Public Health, The University of Tokyo, Tokyo, Japan; e TXP Medical Co., Ltd., Tokyo, Japan

**Keywords:** Prevertebral space infection, Meningitis, Vertebral osteomyelitis, Neck stiffness

## Abstract

While neck stiffness belongs to the classic triad of meningitis symptoms together with fever and altered mental status, it can also be attributed to inflammation from prevertebral space infection. We describe a difficult-to-diagnose case of prevertebral abscess associated with meningitis. Prevertebral space infection, vertebral osteomyelitis, and meningitis are reported to be associated with each other. When a patient presents with an altered mental status due to meningitis, signs and symptoms may be obscured and physicians may be unable to conduct detailed physical examinations or identify symptoms beyond neck stiffness. The threshold for imaging evaluation may need to be lowered for patients at high risk for prevertebral abscess or vertebral osteomyelitis. Physicians need to recognize this clinical entity, as prompt referral to specialists in head and neck surgery is essential for timely drainage.

## Introduction

Prevertebral abscess is one of the serious complications of a prevertebral space infection. The prevertebral space lies between prevertebral fascia anteriorly and vertebral bodies posteriorly, and contains prevertebral muscles and fat. The most common presenting symptom of a cervical prevertebral space infection is neck pain. While other symptoms such as odynophagia, dysphagia, neck stiffness, fever, and back pain are reported, these symptoms are not always present [1]. Physical examination may be unremarkable, though a bulge develops in the oropharynx in some cases. We present a case of difficult-to-diagnose prevertebral abscess.

## Case report

### Initial admission to a community hospital (before transfer)

A 72-year-old man receiving intermittent hemodialysis for diabetic nephropathy presented to a community hospital with fever, walking difficulty, fatigue, and slurred speech. He was diagnosed with pneumonia based on pulmonary infiltration on a chest CT scan and admitted for in-patient care. No blood or sputum culture was obtained. After receiving ceftriaxone as an inpatient for 4 days, he gradually began to experience a disturbance of consciousness and was transferred to our hospital (tertiary emergency hospital) for detailed examination and intensive care (day 1).

### Clinical course at our hospital

On arrival to our emergency department, he was agitated and slightly febrile (37.5 ℃). He presented with a blood pressure of 196/77 mm Hg, pulse of 104/min, and respiratory rate of 24/min with an O2 saturation of 99 % with 2 L of supplemental oxygen. His neck was stiff, but no focal neurologic deficits were noted. His left forearm, the site of an arteriovenous fistula for intermittent dialysis, showed no signs of infection such as warmth, redness or swelling. We were unable to perform further physical examinations, as he was too agitated to cooperate. Bloodwork showed a high white cell count of 145 × 10^9^/L (normal 33–86 × 10^9^/L), high neutrophils of 87.6% (normal 38.5–80.5 %) and high C reactive protein of 40.5 mg/dl (normal < 0.14 mg/dl). Cerebrospinal fluid (CSF) studies showed an elevated leukocyte count of 207 × 10^6^ cells/L (73 % neutrophils, 27 % monocytes), high glucose of 153 mg/dl (normal 50–75 mg/dl), and protein of 108 mg/dl (normal 15–45 mg/dl).

**Fig. 1:**
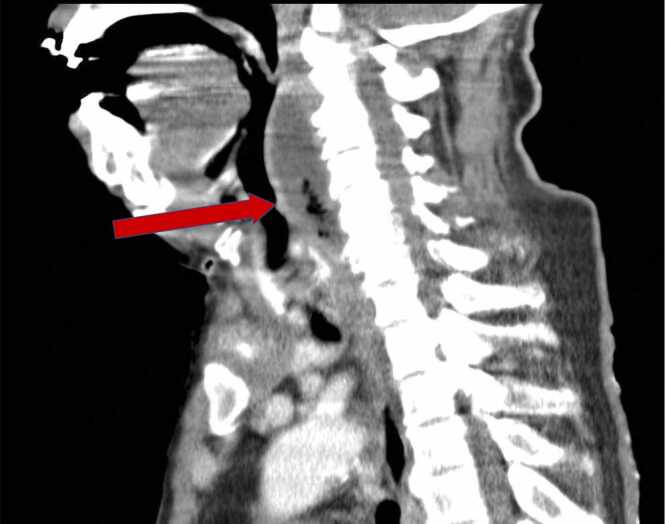
Sagittal view of an enhanced CT showing a region of fluid retention with gas in front of the cervical spine, with an appearance consistent with a retropharyngeal or prevertebral abscess (red arrow).

Judging from the CSF studies, we first considered meningitis to be the cause of his altered mental status. We were concerned, however, by the high risk of blood stream infection (e.g., end stage renal disease), the worsening clinical course (no improvement despite the use of ceftriaxone), and the high inflammatory marker in the bloodwork. Because of the patient’s altered mental status, we were unable to ask him about his subjective symptoms or conduct detailed physical examinations. We performed a whole body enhanced computed tomography scan to rule out infections or abscesses at other sites. The CT scan showed a region of fluid retention with gas in front of the cervical spine, with an appearance consistent with a retropharyngeal or prevertebral abscess (Fig. 1).

**Fig. 2:**
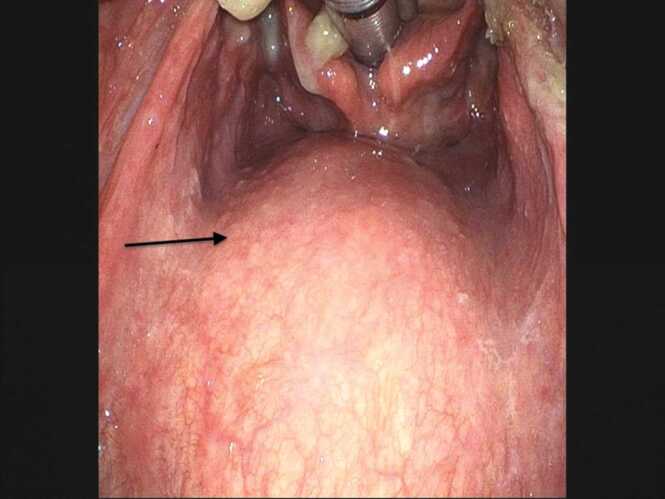
Swelling of the retropharyngeal space on fiberoptic laryngoscopy (black arrow).

An otolaryngologist team performed emergent surgical drainage. The supraglottic fiber showed swelling of the retro-laryngeal space (Fig. 2). The abscess cavity was approached from the right cervical region. Intraoperative findings showed bulging of the prevertebral fascia and abscess formation posteriorly. Incision and drainage were performed and the lesion was diagnosed as a prevertebral abscess.

Cultures of blood, CSF, and the intraoperative specimen were positive for methicillin-resistant *Staphylococcus aureus* on day 2. A transthoracic echocardiogram to evaluate for valve vegetation was negative, which made infectious endocarditis less likely. We treated the patient with antibiotics (meropenem and vancomycin) and switched to vancomycin monotherapy for antibiotic de-escalation based on the culture results. His consciousness gradually improved. Osteomyelitis of the fifth and sixth cervical vertebra appeared on MRI on day 4, but was treated conservatively with antibiotics. Blood culture came back negative on day 36. The patient did not recover functional independence, but regained verbal communication skills. He was transferred to another hospital for rehabilitation and antibiotic treatment on day 46.

## Discussion

We described a case of prevertebral abscess with bacterial meningitis and vertebral osteomyelitis. Vertebral osteomyelitis has reported associations with meningitis and prevertebral abscess [2], [3], [4], and a case report of prevertebral abscess and meningitis has also been published [5]. Neck stiffness can be attributed to either meningeal irritation from meningitis or inflammation from a prevertebral space infection. To our knowledge, this is the first case report of a patient who survived a prevertebral abscess and comorbid meningitis with timely diagnosis and prompt surgical drainage.

The risk factors of prevertebral space infection are not well-known, though evidence suggests they are the same as those of vertebral osteomyelitis: intravenous drug use, alcohol use disorder, liver cirrhosis, hemodialysis, diabetes mellitus, rheumatic disease, immunosuppression, vertebral compression due to malignant metastasis, trauma, disc herniation, infectious endocarditis, and prior surgery [6], [7]. According to a previous study of vertebral osteomyelitis, patients with end-stage renal disease (ESRD) on hemodialysis, diabetes mellitus, liver cirrhosis, malignancy, and infective endocarditis face an increased risk of mortality [7]. In our case, hemodialysis and diabetes mellitus may be the relevant risk factors. The incidence of vertebral osteomyelitis has increased in aging populations [6], [7]. While prevertebral abscess is rare as a complication of prevertebral space infection, it could be an important differential diagnosis among patients with risk factors associated with age.

Various pathogenic microorganisms can cause prevertebral infection or vertebral osteomyelitis, including *Staphylococcus aureus*, Group A *Streptococcus*, *Streptococcus pneumoniae*, and oral anaerobic bacteria [1], [2]. Empiric MRSA coverage may be necessary for patients receiving treatments other conditions (e.g., ESRD patients on hemodialysis), when indicated by the local antibiograms. As we were concerned about meningitis, we selected empiric treatment with meropenem and vancomycin to attain adequate levels of antibiotics in the cerebrospinal fluid. The optimal duration of antibiotic treatment in patients with prevertebral MRSA abscess is unclear. According to a previous study of hematogenous vertebral osteomyelitis, prolonged duration (greater than or equal to 8 weeks) is recommended [8].

Prevertebral abscess and vertebral osteomyelitis are rarely concomitant with bacterial meningitis [2], [5], [9], [10]. The etiology is suggested to be either hematogenous seeding or direct spread via perforation of the dura (vice versa) [2], [5], [10]. In our case, the meningitis was probably caused by a direct spread from adjacent vertebra or hematogenous seeding of *Staphylococcus aureus*. Our patient was unable to verbally express any complaints of neck pain or other symptoms, as he presented to the emergency department with altered mental status due to comorbid meningitis. His consciousness was so severely impaired, he could neither open his mouth nor easily point out the bulge in his posterior pharyngeal wall on physical examination. For these reasons, we had difficulty in promptly diagnosing the prevertebral abscess early in the course of management. Once a patient is diagnosed with meningitis, cognitive biases may make it difficult to find other sources of infection (the most common cognitive bias responsible for misdiagnosis is “premature closure,” a failure to continue considering reasonable alternatives after an initial diagnosis is reached) [11]. Consequently, the delay of timely drainage could be fatal [5].

In summary, we have described our experience with a case with prevertebral abscess and comorbid meningitis and vertebral osteomyelitis. In vulnerable patients (e.g., ESRD) with suspected meningitis, physicians should be mindful of prevertebral abscess and lower the threshold for imaging evaluation (contrast enhanced CT or MRI).

## Sources of Funding

None.

## Ethical approval

Written informed consent was obtained from the patient for publication of this case report and accompanying images.

## Consent

Written informed consent was obtained from the patient for publication of this report and accompanying images. A copy of the written consent is available for review by the Editor-in-Chief of this journal on request.

## CRediT authorship contribution statement

**Hidenori Higashi:** Writing – original draft. **Ken-ichiro Kobayashi:** Writing – review & editing. **Anna Eto:** Writing – review & editing. **Tadahiro Goto:** Writing – review & editing, Supervision.

## Declaration of Competing Interest

None.
